# Historical Records of Mercury Stable Isotopes in Sediments of Tibetan Lakes

**DOI:** 10.1038/srep23332

**Published:** 2016-03-21

**Authors:** Runsheng Yin, Xinbin Feng, James P. Hurley, David P. Krabbenhoft, Ryan F. Lepak, Shichang Kang, Handong Yang, Xiangdong Li

**Affiliations:** 1 State Key Laboratory of Environmental Geochemistry, Institute of Geochemistry, Chinese Academy of Sciences, Guiyang 550002, China; 2 Environmental Chemistry and Technology Program, University of Wisconsin-Madison, Madison, WI, 53706, USA; 3 Department of Civil and Environmental Engineering, University of Wisconsin-Madison, Madison, WI, 53706, USA; 4 University of Wisconsin Aquatic Sciences Center, Madison, WI, 53706, USA; 5 U.S. Geological Survey, Mercury Research Laboratory, Middleton, WI, 53562, USA; 6 State Key Laboratory of Cryospheric Sciences, Cold and Arid Regions Environmental and Engineering Research Institute, Chinese Academy of Sciences, Lanzhou 730000, China; 7 CAS Center for Excellence in Tibetan Plateau Earth Sciences, Chinese Academy of Sciences, Beijing 100101, China; 8 Environmental Change Research Centre, University College London, Pearson Building, Gower Street, London WC1E 6BT, UK; 9 Department of Civil and Environmental Engineering, The Hong Kong Polytechnic University, Hung Hom, Kowloon, Hong Kong

## Abstract

The Tibetan Plateau (TP), known as the “Third Pole”, is a critical zone for atmospheric mercury (Hg) deposition. Increasing anthropogenic activities in the globe leads to environmental changes, which may affect the loading, transport and deposition of Hg in the environment. However, the deposition history and geochemical cycling of Hg in the TP is still uncertain. Our records of Hg and Hg isotopes in sediment profiles of the two largest lakes in the TP, Lake Qinghai and Nam Co, show increased Hg influx since last century, with the maximum Hg influx enrichment ratios of 5.4 and 3.5 in Lake Qinghai and Nam Co, respectively. Shifts in negative δ ^202^Hg in Lake Qinghai (−4.55 to −3.15‰) and Nam Co (−5.04 to −2.16‰) indicate increased atmospheric Hg deposition through rainfall, vegetation and runoff of soils. Mass independent fractionation of both even-Hg (∆ ^200^Hg: +0.05 to +0.10‰) and odd-Hg (∆ ^199^Hg: +0.12 to +0.31‰) isotopes were observed. Positive Δ ^200^Hg suggest high proportion of precipitation-derived Hg in the TP, whereas the positive Δ ^199^Hg results from Hg(II) photo-reduction. Both lakes show increasing Δ ^199^Hg since the 1900 s, and we conclude that with the decrease of ice duration, Hg(II) photo-reduction may have been accelerated in these TP lakes.

The Tibetan Plateau (TP), with an area of ~2.5 × 10^6^ km^2^ and an average elevation of >4000 m above sea level, covers ~26% of land and <1% of population in China. Due to remote location and low population density, the TP is minimally impacted by local anthropogenic activities. However, increasing global industrialization has accelerated the loading, transport and deposition of volatile pollutants (e.g., persistent organic pollutants) to the TP^1^. Like persistent organic pollutants, mercury (Hg) is a globally distributed semi-volatile pollutant and exhibits similar patterns of cycling in the atmosphere^2^, with growing evidence that alpine regions act as intensive sinks of atmospheric Hg^2^,^3^. High atmospheric Hg deposition rates and elevated Hg levels in snow have been reported in the TP^4^,^5^. China and India are two of the largest anthropogenic Hg emission countries to the TP and other sites of the world^6^. Mercury deposited to the watershed and lakes is susceptible to methylation to the bioaccumulable neurotoxin, methylmercury (MeHg)^7^. High levels of MeHg in various aquatic species in TP lakes have been shown, indicating high environmental risks of Hg in this region^7^.

Global industrialization not only cause increasing Hg deposition, but also result in environmental changes. Mercury is a redox-sensitive metal, and its biogeochemical cycling is particularly susceptible to environmental changes^8^,^9^,^10^,^11^. Growing evidence suggests that montane regions experience more rapid environmental changes than lower elevations^12^. For instance, the TP has warmed over the past several decades at twice the global average rate^13^. This has resulted other dependent changes such as cryospheric systems and hydrological regimes^12^,^13^,^14^, which are particularly important to Hg biogeochemical cycling because aquatic environments are active sites of Hg methylation and bioaccumulation^7^,^8^,^9^,^10^,^11^.

Mercury has seven natural stable isotopes (196, 198, 199, 200, 201, 202 and 204), and our understanding of environmental fate of Hg has been enhanced by recent application of Hg isotope geochemistry. In the environment, Hg can undergo both mass dependent fractionation (MDF) and mass independent fractionation (MIF). MDF occurs during a variety of chemical, physical, and biological processes, and has been used to better understand the processes controlling Hg transport, transformation and bioaccumulation^15^,^16^. MIF signatures can provide a unique fingerprint of specific chemical pathways, such as photochemical reactions^17^,^18^. Large variations of both Hg-MDF and -MIF signatures have been documented in different environmental compartments^16^, and can provide multi-dimensional information to identify the sources and better understand biogeochemical Hg cycling^15^.

Sediment profiles coupled with high resolution dating (e.g., ^210^Pb and ^137^Cs) have been broadly used to evaluate historical changes of Hg deposition rate^5^. As the “water tower of Asia”, the TP provides an ideal site to reconstruct environmental changes due to its sensitivity to environmental change and the lack of local pollution sources^13^,^14^. In this study, sediment profiles collected from two of the largest lakes in the TP (Lake Qinghai and Nam Co) were age-dated and analyzed for total Hg concentration (THg) and Hg isotopic composition. The objectives of this study were (1) to elucidate the history of Hg influx and source changes in the TP, and (2) to investigate the influence of global change on the biogeochemical cycle of Hg in this fragile alpine ecosystem.

## Experimental section

### Study area and sampling

**Figure 1:**
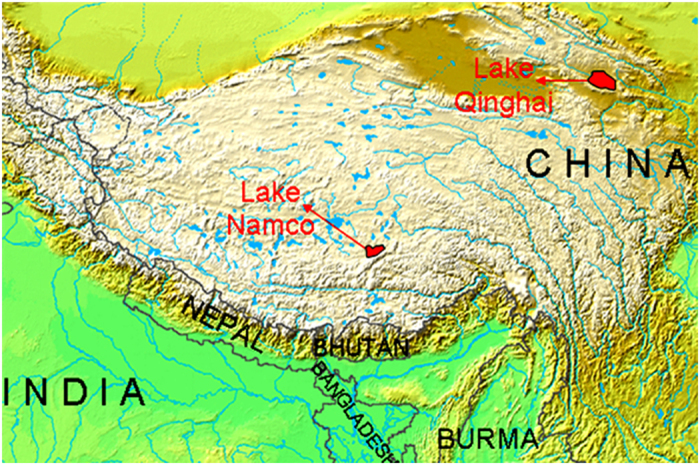
Study area and sampling sites. (This image is modified by R-S Yin, on basis of the a Wikimedia Commons map: https://commons.wikimedia.org/wiki/File:Topografic_map_of_Tibetan_Plateau.png#filelinks).

Lake Qinghai (3194 m), the largest lake (4382 km^2^) in the TP, is located in the northeast of the plateau. Nam Co (4730 m), the second largest lake (1920 km^2^) in the TP, is centrally located (Fig. 1). Lake Qinghai is fed from a catchment of ~29,660 km^2^, and Nam Co has a catchment area of ~15,000 km^2^. The mean annual precipitations in Lake Qinghai and Nam Co are 357 and 414 mm^19^,^20^,^21^,^22^. The present day climate in both lakes is influenced by the Asian monsoon with dry winters, and precipitation mainly occurring in the summer season. The glaciated area of the catchment of Lake Qinghai and Nam Co is ~10 km^2^ and 197 km^2^, respectively, accounting for 0.03% and 1.31% of the catchment. Hence, hydrologic sources to both lakes mainly consist of precipitation, not glacial melt^14^.

Sediment cores were taken from the deepest regions of Lake Qinghai (depth: 25.3 m) in 2006 and Nam Co (depth: ~60 m) in 2009 using HTH gravity corers. The Lake Qinghai core was sectioned in the field using a stainless steel slicer at 0.5 cm intervals from the surface to 5 cm, and then at 1.0 cm intervals to the base of the core. The Nam Co core (21 cm) was sectioned using a stainless steel slicer at 0.5 cm intervals from the surface to the base of the core. Samples were freeze-dried and homogenized prior to ^210^Pb dating, total organic contents (TOC), THg and Hg isotope measurements. Sedimentation rate and TOC methodologies have been reported by Lami *et al.*^20^ and Li *et al.*^22^.

### Hg concentration analysis

THg in sediments was analyzed by direct combustion and atomic absorbance detection based on Lepak *et al.*^23^ at the USGS Wisconsin Mercury Research Lab. SRM (IAEA SL 1) recoveries were within 90~110%, and coefficients of variation of triplicate analyses were less than 10%.

### Mercury isotopic composition analysis

Based on the measured THg concentration [Table S1 of Supplementary Information (SI)], samples and certified reference materials (NIST 2711 and MESS-2) were digested and diluted prior to isotopic measurement on a Neptune Plus MC-ICP-MS housed at the University of Wisconsin-Madison’s State Laboratory of Hygiene. A more detailed method description for MC-ICP-MS analyses is given in the SI. Following the convention recommended by Blum and Bergquist^24^, Hg-MDF is expressed in δ ^202^Hg notation in units of permil (%), referenced to the NIST-3133 Hg standard (analyzed before and after each sample):


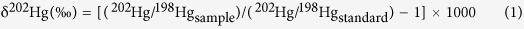


Hg-MIF is reported in Δ notation (Δ ^xxx^Hg) and describes the deviation from mass dependency in units of permil (‰). MIF is the difference between the measured δ ^xxx^Hg and the theoretically predicted δ ^xxx^Hg value using the following formula^24^.


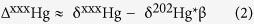


where β is the independent isotope-specific scaling factor determined by the laws of MDF, which 0.2520 for ^199^Hg, 0.5024 for ^200^Hg, and 0.7520 for ^201^Hg.

UM-Almadén solutions^23^ were also measured as 10% of the samples. Data uncertainty reported in this study reflects the larger value of either the external precision of replication of UM-Almadén or the measurement uncertainty of standard reference materials. The overall average and uncertainty of all UM-Almadén measurements (δ ^202^Hg: −0.50 ± 0.04‰; Δ ^199^Hg: −0.03 ± 0.03‰; Δ ^200^Hg: +0.02 ± 0.03‰, σ, n = 9) agreed well with previous results^23^. Measurements of replicate digests of NIST 2711 (δ ^202^Hg: −0.21 ± 0.05%; Δ ^199^Hg: −0.17 ± 0.03%; Δ ^200^Hg: +0.01 ± 0.03‰, σ, n = 3) and MESS-1 (δ ^202^Hg −1.95 ± 0.05‰; Δ ^199^Hg +0.01 ± 0.03‰; Δ ^200^Hg: +0.04 ± 0.03‰, σ, n = 3) were also comparable with previous studies^23^,^25^,^26^,^27^.

## Results and Discussion

### Mercury concentration profiles

**Figure 2:**
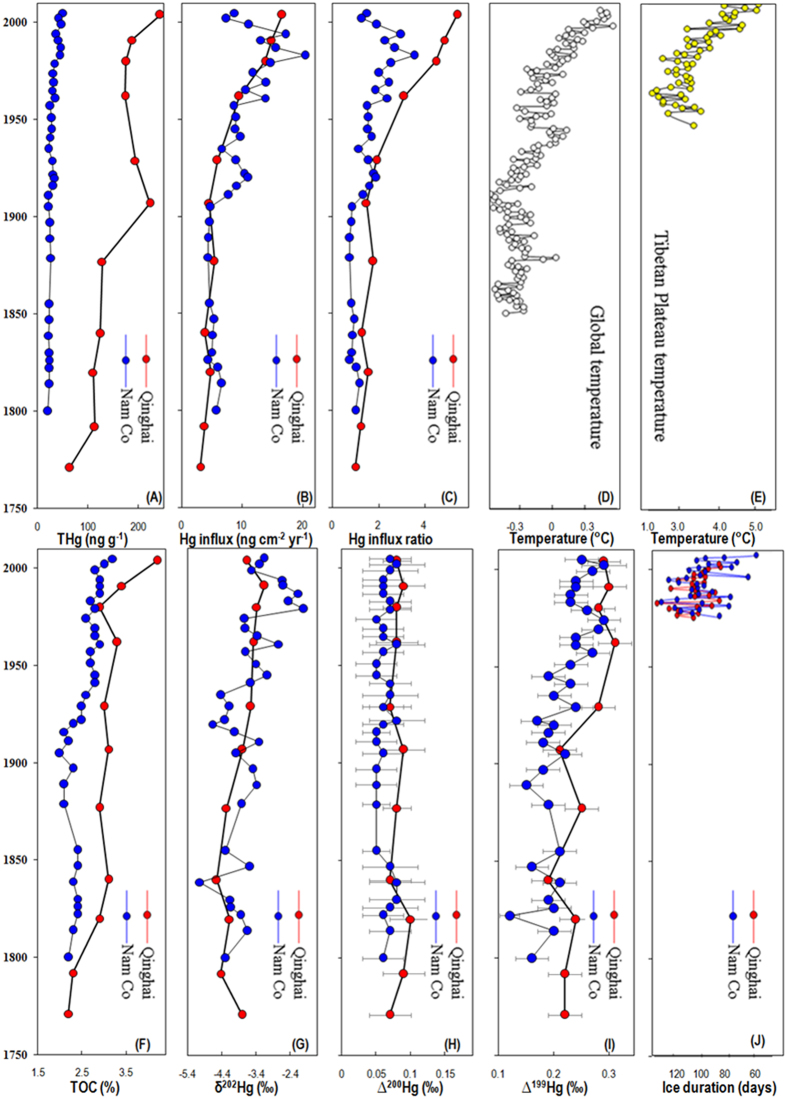
Sediment profiles of THg (**A**), Hg influx (**B**), Hg influx ratio (**C**), global average temperature anomaly ((**D**) according to Hansen *et al.*^30^) and temperature in TP ((**E**), according to Zhang *et al.*^13^), TOC ((**F**) according to Lami *et al.*^20^ and Li *et al.*^22^), δ ^202^Hg (**G**), ∆ ^200^Hg (H), ∆ ^199^Hg (**I**) and ice duration ((**J**) according to Che *et al.*^19^ and Ke *et al.*^21^) in Lake Qinghai and Nam Co.

Historical sediment profiles from both Lake Qinghai and Nam Co show a general trend of increasing THg over the past century (Fig. 2A). THg in sediment provides insight into pollution status, however, influx rates of Hg (sedimentation rate × THg) provide the best estimates of inputs of Hg to lake (Fig. 2B). Preindustrial influxes of Hg in Lake Qinghai and Nam Co are about 3.1 and 5.7 ng cm^−2^ yr^−1^, respectively, with the highest Hg influxes in Lake Qinghai and Nam Co at 16.5 and 20.3 ng cm^−2^ yr^−1^. Mercury influxes among remote lakes have shown to be positively correlated to ratios of terrestrial catchment area (A_C_) to lake area (A_L_)^28^. Nam Co has an A_C_/A_L_ of 7.8 higher than Lake Qinghai (A_C_/A_L_ = 6.7). Mercury influx profile shifts are more clearly evident by calculating influx ratios (influx_sample_/influx_background_, influxes of each sediment with respect to the geochemical background) (Fig. 2C). Influx_sample_ and influx_background_ were the Hg influx of a given sample and the deepest sediment sample in each core, respectively. The maximum influx ratios of Hg in the 21st century are about 5.4 in Lake Qinghai and 3.5 in Nam Co, consistent with other studies of remote lake sediment cores, where Hg influxes have increased by a factor of 3 to 5 compared to the pre-industrial values^5^,^29^. Both profiles indicate increased Hg deposition starting from the early 1900 s, with especially intensive increases since the 1960 s (Fig. 2 A–C). This is in agreement with the increased enhanced global Hg emission (especially China and India) and atmospheric Hg concentrations during the last few decades^5^.

**Figure 3:**
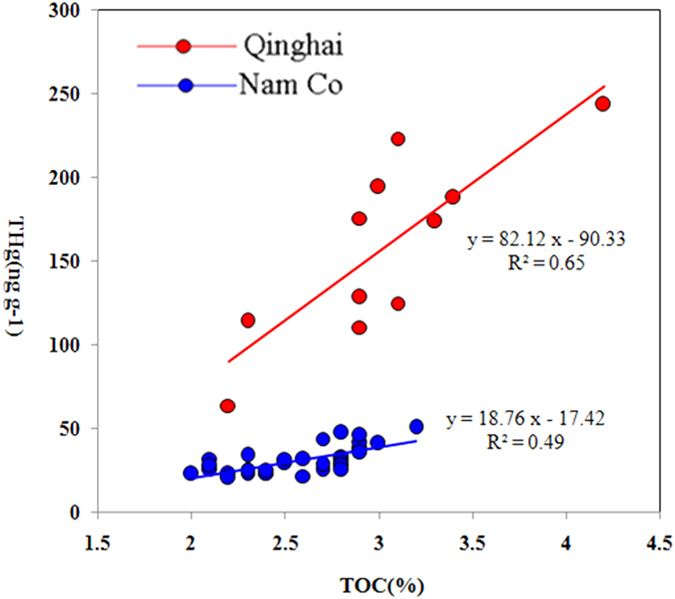
Correlations between THg and TOC in sediments of Lake Qinghai and Nam Co.

The increase of Hg influx in Lake Qinghai and Nam Co is also likely synchronous with the rising global temperature, which starts in the early 1900 s, and has accelerated since the 1960 s (Fig. 2D)^30^. Temperature increase in the TP is twice as high as the global average from 1957 to 2012 (0.036 ± 0.003 °C yr^−1^) (Fig. 2E). This has not only caused increased precipitation at an average annual rate of 10.9 mm per decade from 1961 to 2008, but also resulted in continuous increases of growing season (~1.04 day y^−1^)^31^ and vegetation coverage (3 961.9 km^−2^ yr^−1^)^32^ during the past 2 to 3 decades. Precipitation and vegetation (litterfall) are efficiently scavengers of atmospheric Hg^2^,^4^. Increased precipitation have also caused lake expansion and enhanced soil erosion in the TP^14^. For instance, Nam Co expanded by 20.2% in area between 1976 and 2010, and an average depth increase of 0.11 m^−1^ yr^−1^ was observed in Lake Qinghai in recent years^14^. Enhanced soil erosion in Lake Qinghai and Nam Co during the past few decades has been verified by inert tracers (such as Ti, Ni, Al, Fe, etc)^33^,^34^,^35^. Precipitation and vegetation (litterfall) are important inputs of Hg to pristine soils. The TP is mostly covered by typical alpine meadow and steppe^31^. Increase of plant production in the TP resulted in the increase of soil organic carbon density (0.1 g C m^−2^ yr^−1^) during 1981 to 2010^36^. Organic matter has a strong affinity for Hg^37^. Runoff of organic soil particles may effectively capture vegetation and precipitation-derived Hg from soils and the water column, and ultimately sequester it into sediments^37^,^38^. A recent study observed that organic matter (OM), in sediments of Lake Qinghai is primarily (80%) of terrestrial origin^39^. Significant linear correlations between THg and TOC (P < 0.01, ANOVA test) were observed in Lake Qinghai and Nam Co (Fig. 3). Overall, we suggest that increased anthropogenic Hg emission, enhanced atmospheric Hg deposition (through precipitation and vegetation) and soil erosion, may result in the increased Hg accumulation in the TP lakes.

### Mass dependent fractionation of mercury isotopes

Sediments from Lakes Qinghai and Nam Co showed highly variable δ ^202^Hg values, ranging from −4.55 to −3.15‰ and from −5.04 to −2.16‰, respectively (Fig. 2G), which are much lower than previously reported data for industrial point Hg sources^16^,^25^,^26^,^27^,^40^,^41^,^42^,^43^,^44^,^45^ (δ ^202^Hg: −1.5 to 0‰), consistent with the fact that Lakes Qinghai and Nam Co are less impact by local point sources. Our data are more similar to sediments collected from pristine regions (δ ^202^Hg: −2 to −3‰)^42^,^45^, which mainly receive Hg from atmospheric deposition. Previous studies have reported much higher δ ^202^Hg (0 to +1.0‰) in Hg^0^_g_ collected from pristine sites^46^ in comparison with that collected from urban-industrial regions (δ ^202^Hg: −3 to −0‰)^47^,^48^,^49^. This indicates that Hg with lower δ ^202^Hg values may be preferentially removed during long range transport and deposition through precipitation and litterfall. Indeed, precipitations in Northern America have shown more negative δ ^202^Hg (−4 to 0‰) than that of Hg^0^_g_ (δ ^202^Hg: −0.5 to +1.0‰) in the same areas^46^,^49^,^50^,^51^. For instance, precipitations from urban-industrial regions have shown highly negative δ ^202^Hg values of down to −4.27‰ in China^52^ and −4.37‰ in Florida^50^, being similar to our data of TP sediments. Due to close to China and India, it is possible for such highly fractionated rain contributions to the TP. However, our knowledge about the Hg isotope signatures in precipitation of the TP is limited to one single precipitation event, representing large variabilities^53^. Previous studies also reported negative δ ^202^Hg of −4 to −1‰ for plants, demonstrating that lighter Hg isotopes are preferentially binding within the foliage^46^,^47^,^54^. Increased atmospheric Hg deposition through rainfall and litterfall have caused soils in montane regions to have much negative δ ^202^Hg values^2^. Our observation of negative δ ^202^Hg values is consistent with the fact that atmospheric deposition is the main input of Hg to Lake Qinghai and Nam Co^5^.

In addition to the source effect, post-depositional processes in the water column may also affect δ ^202^Hg in sediments. Mercury deposited into lakes can be re-emitted to the atmosphere, while the remaining fraction is adsorbed on and settled by sediment particulate. The product Hg(0) during volatilization, microbial reduction and photoreduction processes could result in more negative δ ^202^Hg values and, likewise, the residual Hg in the water column will result in a more positive δ ^202^Hg values^17^,^18^,^55^,^56^,^57^. Adsorption of aqueous Hg(II) by sediment particles containing thiol groups^58^, goethite^59^ and sulfides^60^ is likely cause negative shifts of δ ^202^Hg (−0.60‰) in the solid phase. However, significant shift of δ ^202^Hg may only occur when very small fraction of Hg is adsorbed relative to the total Hg in a system. Given the fact that particulate Hg is the dominate form of total Hg in Nam Co (86.7%) and other TP lakes^61^,^62^, we would not expect a significant negative shift of δ ^202^Hg during adsorption of aqueous Hg(II) by sediments.

**Figure 4:**
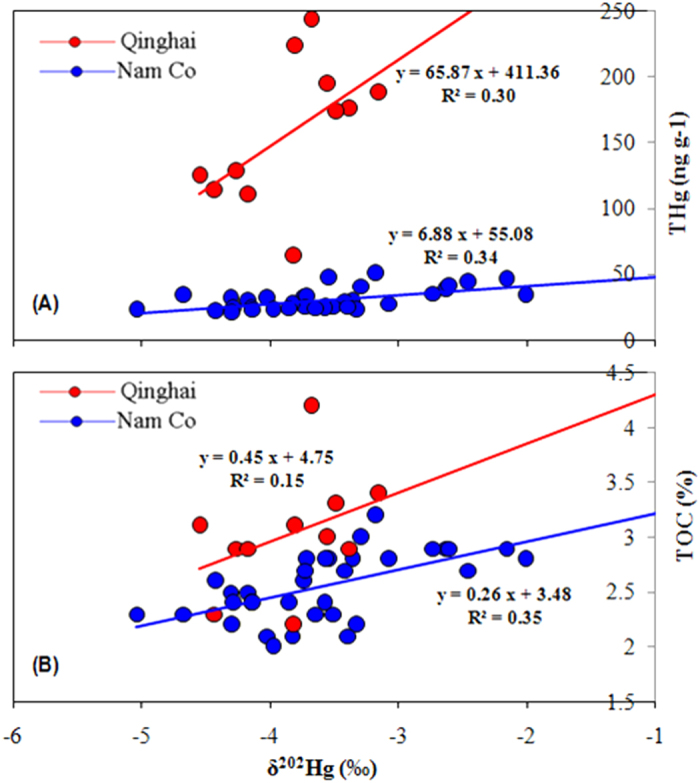
Relations of δ 202Hg to THg (**A**) and TOC (**B**) in sediments of Lake Qinghai and Nam Co.

Like the THg profiles, δ ^202^Hg generally increases from the deep part to the surface layer of the two cores (Fig. 2G). This pattern is similar to sediment cores collected near anthropogenic Hg point sources, where increased inputs of anthropogenic Hg with δ ^202^Hg ranges from −1 to 0‰ have shown in upper layer sediments^25^,^43^,^44^. It is unclear whether the increase of δ ^202^Hg in this study is the result of global anthropogenic Hg input, however, due to the sparse population and industrial activities within the TP, local point sources may not explain the significant δ ^202^Hg increase upcore. The shift of δ ^202^Hg may be explained by a combined effect of enhanced precipitation, net primary production and soil erosion, all of which could incorporate more isotopic heavier Hg^0^_g_ into waters, soils and sediments. Significant correlations between δ ^202^Hg and THg with TOC were observed in Nam Co (*P* < 0.01, ANOVA test), when compared to that in Lake Qinghai (Fig. 4A,B). This suggests that the shifts of δ ^202^Hg in Nam Co are more influenced by input of precipitation and vegetation derived Hg, and runoff of organic soils, as supported by the smaller lake area (1920 km^2^) of Nam Co. The lower correlation between δ ^202^Hg and THg (*P* > *0.05*) with TOC (*P* > *0.05*) in Lake Qinghai may indicate sediments more influenced by lake processes, with a the much large lake area (4382 km^2^).

### Mass independent fractionation of ^200^Hg

In this study, small but detectable MIF of ^200^Hg was found in Lake Qinghai (∆ ^200^Hg: +0.07 to +0.10‰) and Nam Co (∆ ^200^Hg: +0.05 to +0.08‰) (Fig. 2H). When compared to the analytical uncertainty for ∆ ^200^Hg (±0.03‰), these results are considered significant. ∆ ^200^Hg values of sediments from both Lake Qinghai and Nam Co were also significantly higher (*P* < *0.01*, T-test) than UM-Almadén. The mechanism for MIF of ^200^Hg is still unclear; however, prior studies have suggested that ^200^Hg MIF is likely linked to photo-initiated Hg^0^_g_ oxidation^49^,^51^. Significant ^200^Hg MIF has been reported in atmospheric Hg samples^46^,^48^,^49^,^50^,^51^,. In general, Hg^0^_g_ is characterized by negative ∆ ^200^Hg (−0.4 to 0‰), whereas precipitation (containing oxidized Hg species) is characterized by positive ∆ ^200^Hg (+1.2 to 0‰)^46^,^48^,^49^,^50^,^51^. Industrial Hg has shown to be absent of ^200^Hg MIF (∆ ^200^Hg: ~0‰)^16^,^26^,^27^,^40^. MIF of ^200^Hg has not been reported in soils and sediments from urban-industrial regions^41^,^42^,^43^,^44^,^45^, most likely a result of the dilution effect by industrial Hg. However, vegetation can incorporate atmospheric Hg^0^_g_, and therefore have shown slightly negative ∆ ^200^Hg (mean: −0.10‰, n = 5) in Northern Sweden forests^54^. A recent study also reported pronounced positive ∆ ^200^Hg in sediments collected from the Laurentian Great Lakes where precipitation is the major Hg input to sediments^23^. Our observation of positive ∆ ^200^Hg may highlight the importance of precipitation Hg in TP lakes.

If precipitation ∆ ^200^Hg signature did not change over time in the TP, increases of ∆ ^200^Hg in sediment profiles result from enhanced precipitation Hg inputs are expected. However, we observed the consistent ∆ ^200^Hg pattern in both Lake Qinghai and Nam Co (Fig. 2H). The lack of increased ∆ ^200^Hg with elevated precipitation rates in this study may be explained by the isotope dilution of ∆ ^200^Hg by other sources. The magnitude of ∆ ^200^Hg in precipitation have shown to decrease from pristine to urban-industrial regions^49^,^50^,^51^, suggesting the dilution by industrial Hg (∆ ^200^Hg: ~0‰)^16^,^26^,^27^,^40^. As mentioned earlier, enhanced input of soil- and vegetation-derived Hg with negative to zero ∆ ^200^Hg, may also lessen the increase of ∆ ^200^Hg in sediments. However, due to the lack of Hg isotope data in precipitation and soils throughout time, assessment of Hg contributions from precipitation and soil erosion was not performed in our study.

### Mass independent fractionation of ^199^Hg and ^201^Hg

**Figure 5:**
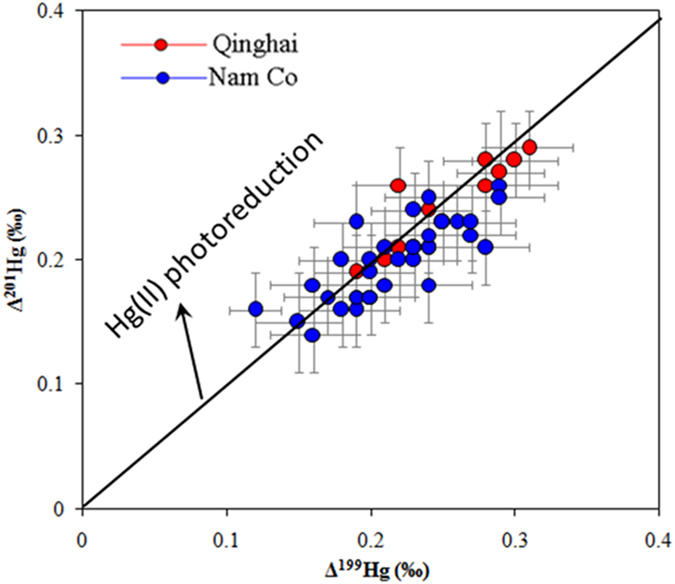
Relations between ∆ ^199^Hg and ∆ ^201^Hg in sediments of Lake Qinghai and Nam Co.

Positive MIF of odd Hg isotopes (^199^Hg and ^201^Hg) was observed in sediment of both lakes (Fig. 2I). The ∆ ^199^Hg values in Lake Qinghai and Nam Co range from +0.19 to +0.31‰ and +0.12 to +0.28‰, respectively. There are two known possible mechanisms for odd-MIF: the nuclear volume effect (NVE)^63^ and the magnetic isotope effect (MIE)^17^. Laboratory experiments demonstrated that NVE can be caused during elemental Hg^0^ volatilization^64^, equilibrium Hg-thiol complexation^58^ and dark Hg(II) reduction^57^ with ∆ ^199^Hg/∆ ^201^Hg of ~1.6. Effects on MIE are mainly due to the photoreactions of aqueous Hg species in the presence of dissolved organic carbon (DOC), resulting in ∆ ^199^Hg/∆ ^201^Hg of 1.00~1.30^17^,^18^. This is comparable with the observed ∆ ^199^Hg/∆ ^201^Hg ratio (1.07 ± 0.07, 2σ) in all sediments investigated in this study, suggesting that aqueous Hg(II) photo-reduction is the possible process to cause MIF of Hg isotopes (Fig. 5).

The positive ∆ ^199^Hg in TP lake sediments is different from previous data on sediments collected from industrial-urban regions, which mainly have negative to zero ∆ ^199^Hg^27^,^41^,^43^,^44^,^45^. Industrial Hg sources have shown average ∆ ^199^Hg close to zero^16^,^40^,^41^,^43^, and continental soils and vegetation mainly showed negative ∆ ^199^Hg values (−0.5 to 0‰)^2^,^26^,^46^,^47^,^54^. The positive ∆ ^199^Hg of the TP sediments may be explained by the inputs of Hg with positive ∆ ^199^Hg or Hg(II) photoreduction in the water column, or both. Positive ∆ ^199^Hg values (0 to +1.0‰) have been reported for precipitation collected from different sites of the world^49^,^50^,^51^,^52^,^53^. Interestingly, sediment cores in this study reflect a shift of +0.1‰ in Δ ^199^Hg values since the early 1900 s (Fig. 2I), three times higher than our analytical uncertainty for UM-Almadén (Δ ^199^Hg: ±0.03‰, σ). Increased precipitation tends to cause rise of ∆ ^199^Hg in sediments, however, it also results in more input of vegetation- and soil-derived Hg (with negative ∆ ^199^Hg) to lakes, likely to lessen the increase of ∆ ^199^Hg in sediment profiles. Like the consistent ∆ ^200^Hg profiles, we would not expect a significant shift of ∆ ^199^Hg due to enhanced inputs of vegetation- and soil-derived Hg.

In this study, increases of Δ ^199^Hg are more likely the result from enhanced Hg(II) photoreduction in the lake water column before incorporation into sediments. Photoreduction of Hg is largely controlled by solar irradiation and water conditions^17^,^18^,^42^,^65^. Long-term observation demonstrated no clear patterns on solar irradiation in the TP^66^. The increased Δ ^199^Hg patterns in both lakes (Fig. 2I) show similar patterns with temperature rising (Fig. 2D,E). Positive relations between Δ ^199^Hg and rising temperatures were observed (Fig. S1 of SI). Temperature rising have caused decreases of ice cover in the TP lakes, which can lead to greater exposure to sunlight for increased photochemical activity^9^,^10^. Ice cover should play an important role in controlling Hg(II) photoreduction in the TP lakes, considering the long-term of the ice duration in the TP lakes. Due to rising temperature, ice duration in the TP lakes have declined (Fig. 2J). Negative linear correlations between temperature and time of ice duration in 36 lakes in the TP has been also observed^13^. We speculate that the thickness of the lake ice would also decline along with rising temperatures, causing more water to be exposed to sunlight.

**Figure 6:**
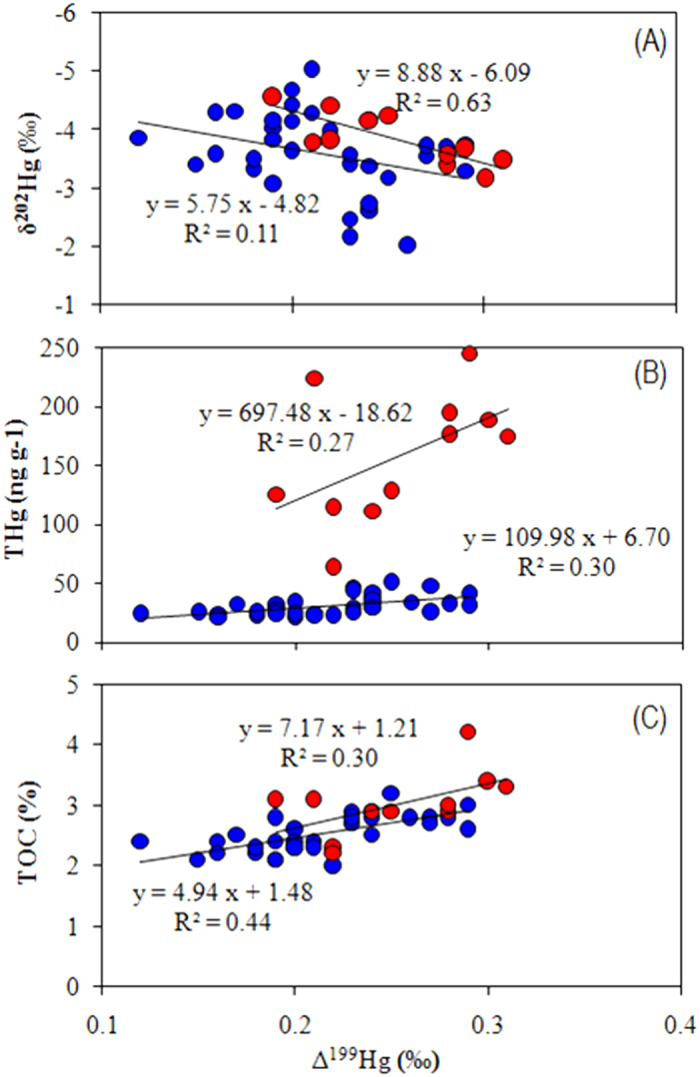
Relations of ∆ ^199^Hg to δ ^202^Hg(**A**), THg (**B**) and TOC (**C**) in sediments of Lake Qinghai and Nam Co.

Positive relationships between ∆ ^199^Hg and δ ^202^Hg (Fig. 6A) were observed in Lake Qinghai (*P* < 0.01) and Nam Co (*P* < 0.01). Laboratory experiments on Hg(II) photoreduction also revealed positive relations between ∆ ^199^Hg and δ ^202^Hg with a δ ^202^Hg/∆ ^199^Hg of 0.83^16^, which is much smaller than that observed for Lake Qinghai (δ ^202^Hg/∆ ^199^Hg = 8.88) and Nam Co (δ ^202^Hg/∆ ^199^Hg = 5.75). This suggests that Hg(II) photoreduction may not be the main cause of the positive shifts of δ ^202^Hg in the TP lakes. The positive relations between ∆ ^199^Hg and δ ^202^Hg indicate that enhanced Hg(II) photoreduction and δ ^202^Hg shifts are induced by similar reasons, possibly due to the temperature effect. Indeed, warming not only causes decrease of ice duration which leads to higher Hg(II) photoreduction, but also results in higher influxes of atmospheric Hg (with higher δ ^202^Hg values) though rainfall and soil erosion into the lakes. Our assumption has been supported by significant positive relations between ∆ ^199^Hg and THg (*P* < 0.01) (Fig. 6B), and TOC (*P* < 0.01) (Fig. 6C) in Nam Co. Like δ ^202^Hg, we also observed less correlation between ∆ ^199^Hg and THg (P > 0.05), and TOC (P > 0.05) in Lake Qinghai, which indicates that Lake Qinghai may be more influenced by in-lake processes. Further research on water column Hg processes of Lake Qinghai are needed to better understand the variations of Hg isotopes in this study.

### Environmental implications

Alpine regions function as important convergence zones for atmospheric Hg, and have a rapid response to environmental change. Environmental changes such as enhanced precipitation, higher terrestrial plant biomass, and erosion of soils, may result in greater atmospheric Hg deposition and transport of historically deposited legacy Hg into the lakes of the in the TP. Dramatic lake ice cover reduction in TP may lead to increased Hg(II) photoreduction and evasion of Hg^0^_(g)_. The results of this study suggest that environmental change signals can be seen in the Hg isotopic distribution in the TP lake sediments. It should be mentioned that increased precipitation and glacier shrink have resulted in lake expansion and flooding of organic soil horizons^67^, which may affect the food web structures, Hg methylation and demethylation rates, and Hg fluxes on sediment-water–atmosphere interfaces of the TP lakes. Further studies are therefore needed.

## Additional Information

**How to cite this article**: Yin, R. *et al.* Historical Records of Mercury Stable Isotopes in Sediments of Tibetan Lakes. *Sci. Rep.* **6**, 23332; doi: 10.1038/srep23332 (2016).

## Supplementary Material

Supplementary Information
