# Supplementary material for: Historical Records of Mercury Stable Isotopes in Sediments of Tibetan Lakes
Source: Sci Rep. 2016 Mar 21;6:23332. doi: 10.1038/srep23332 (PMC4800404; doi:10.1038/srep23332)
Supplement: Supplementary Information [file srep23332-s1.doc]

**Supporting Information**

# Historical Records of Mercury Stable Isotopes in Sediments of Tibetan Lakes

Runsheng Yin^1, 2^, Xinbin Feng^1, *^, James P. Hurley^2^, ^3, 4^, David P. Krabbenhoft^5^, Ryan F. Lepak^2^, Shichang Kang^6, 7^, Handong Yang^8^, Xiangdong Li^9^

^1^*State Key Laboratory of Environmental Geochemistry, Institute of Geochemistry, Chinese Academy of Sciences, Guiyang 550002, China*

^2^*Environmental Chemistry and Technology Program, University of Wisconsin-Madison, Madison, WI, 53706, USA*

^3^*Department of Civil and Environmental Engineering, University of Wisconsin-Madison, Madison, WI, 53706, USA*

^4^*University of Wisconsin Aquatic Sciences Center, Madison, WI, 53706, USA*

^5^*U.S. Geological Survey, Mercury Research Laboratory, Middleton, WI, 53562, USA*

^6^*State Key Laboratory of Cryospheric Sciences, Cold and Arid Regions Environmental and Engineering Research Institute, Chinese Academy of Sciences, Lanzhou 730000, China*

^7^*CAS Center for Excellence in Tibetan Plateau Earth Sciences, Chinese Academy of Sciences, Beijing 100101, China*

^8^*Environmental Change Research Centre, University College London, Pearson Building, Gower Street, London WC1E 6BT, UK*

^9^*Department of Civil and Environmental Engineering, The Hong Kong Polytechnic University, Hung Hom, Kowloon, Hong Kong*

*^*^Contact author: fengxinbin@vip.skleg.cn*

**Experimental Methods**

About 0.5 g of sample was digested (95 °C, 1 h) by 5 mL aqua regia (HCl:HNO_3_ = 3:1, v:v). Certified reference materials (NIST-2711, n=3; MESS-2, n=3) were prepared using the same procedure. Based on the measured THg [Table S1], sample digests of Lake Qinghai were diluted to 1 ng mL^-1^ Hg; Sample digests of Nam Co were diluted to Hg of 0.5 ng mL^-1^, whereas for some low Hg samples (THg < 25 ng g^-1^), they were diluted to Hg of 0.4 ng mL^-1^. All diluted solutions have acid concentration of < 20%. Duplicate UM-Almadén solutions containing 0.3 (n=3), 0.5 (n=3) and 1.0 (n=3) ng mL^-1^ of Hg were prepared into 10% aqua regia. NIST SRM 3133 with different Hg concentrations (0.4, 0.5 and 1.0 ng mL^-1^) were also prepared into 10% aqua regia Tl standard solutions (NIST SRM 997) with Tl concentration of 20 ng g^-1^ were prepared by 0.12M HCl. Acids (HCl and HNO_3_) used here were of ultrapure grades (certified ACS Plus, Fisher Scientific). The 18.2-ΩM-grade water used for the preparation of reagents and solutions was produced by a Milli-Q water system (Millipore, Bedford, USA).

Hg isotopic compositions were measured by a Neptune Plus MC-ICP-MS housed at the University of Wisconsin-Madison’s State Laboratory of Hygiene. The instrument was connected with a gas-liquid phase separator and an Apex-Q desolvation unit (Elemental Scientific Inc., USA) for Hg and Tl introduction, respectively. Briefly, stannous chloride (SnCl_2_ 3% in 10% HCl, w/v) and Hg solutions were continually pumped onto a frosted glass phase separator, producing gaseous elemental Hg(0) which was mixed with the dry Tl aerosol generated by the Apex-Q nebulizer, before being introduced into MC-ICP-MS. The Apex-Q was set in the free flow-mode using the “sample” gas of the MC-ICP-MS, and the glass phase separator was flushed by the “additional” gas of the instrument. Tl (NIST SRM 997, ^205^Tl/^203^Tl = 2.38714) was used as an internal standard for simultaneous instrumental mass bias correction of Hg. Seven of the nine faraday cups, L3, L2, L1, C, H1, H2 and H3, were used to monitor the ^198^Hg, ^199^Hg, ^200^Hg, ^201^Hg, ^202^Hg, ^203^Tl and ^205^Tl isotopes, respectively. The gains of the amplifier associated with each Faraday cup were calibrated for efficiency on a daily basis. Instrument parameters (e.g., Ar gas flows, torch settings, and lens system) were tuned for a maximum ion intensity of Hg and Tl in standard solutions. Operating conditions and instrumentation of the present study are given in Table S2. Data were acquired by 3 blocks (60 cycles per block), monitoring all isotopes for 2.097 s per cycle. An initial uptake of Hg solution of 3 min were programmed to ensure stable signals during measurements. Between measurements, the glass phase separator was washed by 0.8 M HNO_3_ for about 6 min until the intensities of Hg returned to background level. High sensitivity X skimmer cone and Jet sample cone were used. The intensities for ^202^Hg and ^205^Tl were <0.02 V for acid blanks. The sensitivity for ^201^Hg during analysis is estimated to be 0.6 to 0.7 V per ng mL^-1^ Hg, and the intensities of ^205^Tl for 20 ng mL^-1^ Tl standard solution during all analysis range from 3.7 to 4.0 V.

A standard-sample-standard bracketing method was used to compare relative per mil (‰) deviation (using the δ notation) of the sample to NIST SRM 3133(Blum and Bergquist, 2007):

δ^xxx^Hg(‰) = {(^xxx^Hg/^198^Hg_sample_)/(^xxx^Hg/^198^Hg_NIST 3133_)－1}×1000 (1)

Hg-MIF is reported in Δ notation (Δ^xxx^Hg, deviation from mass dependency in units of permil) and is the difference between the measured Δ^xxx^Hg and the theoretically predicted Δ^xxx^Hg value using the following equations (Blum and Bergquist, 2007):

Δ^199^Hg ≈ δ^199^Hg - δ^202^Hg*0.2520 (2)

Δ^200^Hg ≈ δ^200^Hg - δ^202^Hg*0.5024 (3)

Δ^201^Hg ≈ δ^201^Hg - δ^202^Hg*0.7520 (4)

UM-Almadén solutions were measured in every 10 samples. According the ^201^Hg signal, The concentrations of Hg in the bracketing NIST SRM 3133 solutions were within 8% of that in the UM-Almadén. THg in acid digests were also monitored by MC-ICP-MS using ^201^Hg signals, the concentration of Hg between sample digests and NIST 3133 were within 6%, which show that the recoveries of Hg in acid digests were >94%. Data uncertainties reported in this study reflect the larger values of either the external precision of the replication of the UM-Almadén or the measurement uncertainty of standard reference materials. No static differences in Hg isotopic compositions were observed for UM-Almadén with different Hg concentrations (Table S3). The overall average and uncertainty of all UM-Almadén measurements (δ^202^Hg: -0.50 ± 0.04‰; Δ^199^Hg: -0.03 ± 0.03‰; Δ^200^Hg: +0.02 ± 0.03‰, σ, n=9) agreed well with Blum and Bergquist (2007). Measurements of replicate digests of NIST 2711 (δ^202^Hg: -0.21±0.05‰; Δ^199^Hg: -0.17±0.03‰; Δ^200^Hg: +0.01 ± 0.03‰, σ, n=3) and MESS-1 (δ^202^Hg -1.95 ± 0.05‰; Δ^199^Hg +0.01 ± 0.03‰; Δ^200^Hg: +0.04 ± 0.03‰, σ, n=3) were also comparable with previous studies (Biswas et al., 2008; Donovan et al., 2013; Yin et al., 2014).

**References**

Biswas A, Blum JD, Bergquist BA, et al., Natural mercury isotope variation in coal deposits and organic soils. *Environ Sci Technol***42**, 8303-8309 (2008)

Blum JD, Bergquist BA. Reporting of variations in the natural isotopic composition of mercury. *Anal Bioanal Chem* **388**, 353-359 (2007)

Donovan PM, Blum JD, Yee D, et al. An isotopic record of mercury in San Francisco Bay sediment. *Chem Geol* **349**, 87-98 (2013)

Hansen, J., et al. Climate simulations for 1880–2003 with GISS model. *Climate Dynamics* **29**, 661-696 (2007)

Yin R, Feng X, Chen J. Mercury stable isotopic compositions in coals from major coal producing fields in China and their geochemical and environmental implications. *Environ Sci Technol* **48**, 5565-5574 (2014)

**Figure S1** Correlations between global average temperature anomaly (Hansen et al. 2007) and ∆^199^Hg values in Lake Qinghai and Nam Co


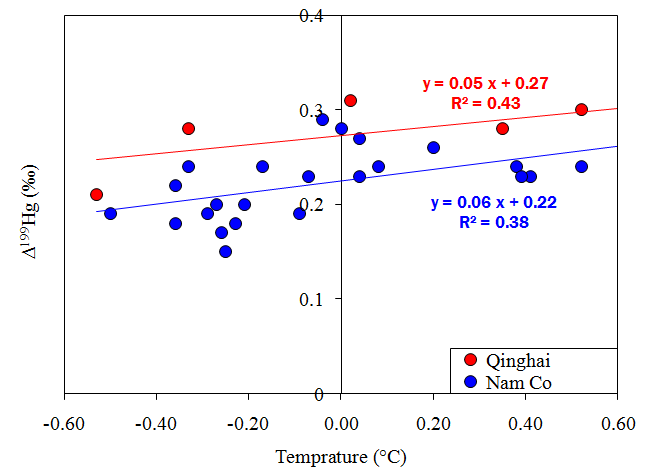


## Table S1 Age, depth, Hg isotope compositions, fraction of Hg(II) photoreduction and total Hg concentrations in sediments of Lake Qinghai and Nam Co

| Sample | Age | Depth | δ^199^Hg | δ^200^Hg | δ^201^Hg | δ^202^Hg | Δ^199^Hg | Δ^200^Hg | Δ^201^Hg | THg | Hg influx | Influx |
| --- | --- | --- | --- | --- | --- | --- | --- | --- | --- | --- | --- | --- |
| ID | (A.D.) | (cm) | ‰ | ‰ | ‰ | ‰ | ‰ | ‰ | ‰ | ng g^-1^ | ng m^-2^ yr^-1^ | ratio |
| **Lake Qinghai** | | | | | | | | | | |  |  |
| QH-1 | 2004 | 0.25 | -0.64 | -1.77 | -2.50 | -3.68 | 0.29 | 0.08 | 0.27 | 243.9 | 16.5 | 5.4 |
| QH-2 | 1991 | 1.5 | -0.49 | -1.49 | -2.09 | -3.15 | 0.30 | 0.09 | 0.28 | 187.9 | 14.8 | 4.8 |
| QH-3 | 1980 | 2.25 | -0.57 | -1.62 | -2.26 | -3.38 | 0.28 | 0.08 | 0.28 | 175.9 | 13.7 | 4.5 |
| QH-4 | 1962 | 3.25 | -0.57 | -1.67 | -2.33 | -3.48 | 0.31 | 0.08 | 0.29 | 173.8 | 9.5 | 3.1 |
| QH-5 | 1929 | 4.5 | -0.61 | -1.71 | -2.41 | -3.55 | 0.28 | 0.07 | 0.26 | 194.4 | 5.8 | 1.9 |
| QH-6 | 1907 | 5.25 | -0.75 | -1.82 | -2.66 | -3.80 | 0.21 | 0.09 | 0.20 | 223.5 | 4.5 | 1.5 |
| QH-7 | 1877 | 6.25 | -0.82 | -2.06 | -2.97 | -4.26 | 0.25 | 0.08 | 0.23 | 128.3 | 5.4 | 1.8 |
| QH-8 | 1840 | 7.5 | -0.96 | -2.22 | -3.23 | -4.55 | 0.19 | 0.07 | 0.19 | 125 | 3.8 | 1.3 |
| QH-9 | 1820 | 8.25 | -0.81 | -2.00 | -2.90 | -4.17 | 0.24 | 0.10 | 0.24 | 110.6 | 4.8 | 1.6 |
| QH-10 | 1792 | 9.25 | -0.90 | -2.14 | -3.07 | -4.43 | 0.22 | 0.09 | 0.26 | 114 | 3.7 | 1.2 |
| QH-11 | 1771 | 10 | -0.74 | -1.84 | -2.66 | -3.81 | 0.22 | 0.07 | 0.21 | 63.8 | 3.1 | 1 |
| **Nam Co** [*Kang et al. *submitted*] | | | | | | | | | | |  |  |
| NMC-1 | 2005 | 0.25 | -0.55 | -1.52 | -2.15 | -3.17 | 0.25 | 0.07 | 0.23 | 50.8 | 8.7* | 1.5 |
| NMC-2 | 2002 | 0.75 | -0.54 | -1.57 | -2.21 | -3.29 | 0.29 | 0.08 | 0.26 | 40.9 | 7.3* | 1.3 |
| NMC-3 | 1999 | 1.25 | -0.62 | -1.71 | -2.44 | -3.54 | 0.27 | 0.07 | 0.22 | 47.5 | 11.0* | 1.9 |
| NMC-4 | 1994 | 1.75 | -0.42 | -1.26 | -1.79 | -2.62 | 0.24 | 0.06 | 0.18 | 37.9 | 17.1* | 3 |
| NMC-5 | 1991 | 2.25 | -0.42 | -1.25 | -1.75 | -2.60 | 0.24 | 0.06 | 0.21 | 42.2 | 13.0* | 2.3 |
| NMC-6 | 1987 | 2.75 | -0.31 | -1.03 | -1.41 | -2.16 | 0.23 | 0.06 | 0.21 | 45.8 | 15.5* | 2.7 |
| NMC-7 | 1983 | 3.25 | -0.39 | -1.16 | -1.60 | -2.45 | 0.23 | 0.07 | 0.24 | 43.9 | 20.3* | 3.5 |
| NMC-8 | 1979 | 3.75 | -0.25 | -0.94 | -1.28 | -2.01 | 0.26 | 0.07 | 0.23 | 33.5 | 14.6* | 2.6 |
| NMC-9 | 1974 | 4.25 | -0.65 | -1.82 | -2.55 | -3.73 | 0.29 | 0.05 | 0.25 | 31.4 | 11.6* | 2 |
| NMC-10 | 1969 | 4.75 | -0.65 | -1.80 | -2.58 | -3.71 | 0.28 | 0.06 | 0.21 | 32.8 | 13.9* | 2.4 |
| NMC-11 | 1965 | 5.25 | -0.61 | -1.63 | -2.32 | -3.36 | 0.24 | 0.06 | 0.21 | 29.1 | 10.6* | 1.8 |
| NMC-12 | 1961 | 5.75 | -0.45 | -1.29 | -1.80 | -2.73 | 0.24 | 0.08 | 0.25 | 35.6 | 13.7* | 2.4 |
| NMC-13 | 1957 | 6.25 | -0.67 | -1.81 | -2.57 | -3.72 | 0.27 | 0.06 | 0.23 | 25.4 | 8.5* | 1.5 |
| NMC-14 | 1951 | 6.75 | -0.63 | -1.66 | -2.36 | -3.41 | 0.23 | 0.05 | 0.20 | 28.2 | 9.0* | 1.6 |
| NMC-15 | 1945 | 7.25 | -0.58 | -1.49 | -2.14 | -3.07 | 0.19 | 0.05 | 0.17 | 27.7 | 8.8* | 1.5 |
| NMC-16 | 1941 | 7.75 | -0.67 | -1.72 | -2.47 | -3.56 | 0.23 | 0.07 | 0.21 | 24.9 | 9.7* | 1.7 |
| NMC-17 | 1935 | 8.25 | -0.91 | -2.15 | -3.13 | -4.42 | 0.20 | 0.07 | 0.19 | 21.5 | 6.5* | 1.1 |
| NMC-18 | 1929 | 8.75 | -0.81 | -2.04 | -2.92 | -4.17 | 0.24 | 0.06 | 0.22 | 29.1 | 8.9* | 1.6 |
| NMC-19 | 1922 | 9.25 | -0.92 | -2.09 | -3.07 | -4.31 | 0.17 | 0.08 | 0.17 | 31.9 | 10.2* | 1.8 |
| NMC-20 | 1920 | 9.75 | -0.98 | -2.29 | -3.34 | -4.67 | 0.20 | 0.06 | 0.17 | 34 | 10.9* | 1.9 |
| NMC-21 | 1916 | 10.25 | -0.82 | -1.97 | -2.86 | -4.02 | 0.19 | 0.05 | 0.16 | 31.8 | 9.0* | 1.6 |
| NMC-22 | 1911 | 10.75 | -0.66 | -1.62 | -2.34 | -3.32 | 0.18 | 0.05 | 0.16 | 22.6 | 7.7* | 1.3 |
| NMC-23 | 1905 | 11.25 | -0.78 | -1.94 | -2.79 | -3.98 | 0.22 | 0.06 | 0.20 | 22.7 | 4.8* | 0.8 |
| NMC-24 | 1897 | 11.75 | -0.70 | -1.71 | -2.43 | -3.50 | 0.18 | 0.05 | 0.20 | 25.5 | 4.6* | 0.8 |
| NMC-25 | 1889 | 12.25 | -0.70 | -1.65 | -2.40 | -3.39 | 0.15 | 0.05 | 0.15 | 25.4 | 4.3* | 0.7 |
| NMC-26 | 1879 | 12.75 | -0.78 | -1.87 | -2.65 | -3.83 | 0.19 | 0.05 | 0.23 | 27.4 | 4.3* | 0.8 |
| NMC-28 | 1855 | 13.75 | -0.87 | -2.10 | -3.04 | -4.28 | 0.21 | 0.05 | 0.18 | 23.5 | 4.6* | 0.8 |
| NMC-30 | 1847 | 14.75 | -0.74 | -1.73 | -2.51 | -3.58 | 0.16 | 0.07 | 0.18 | 23.7 | 5.3* | 0.9 |
| NMC-32 | 1839 | 15.75 | -1.06 | -2.45 | -3.58 | -5.04 | 0.21 | 0.08 | 0.21 | 22.7 | 5.1* | 0.9 |
| NMC-34 | 1830 | 16.75 | -0.86 | -2.00 | -2.95 | -4.15 | 0.19 | 0.08 | 0.17 | 24 | 4.9* | 0.9 |
| NMC-35 | 1826 | 17.25 | -0.84 | -2.00 | -2.91 | -4.13 | 0.20 | 0.07 | 0.20 | 23.3 | 4.3* | 0.8 |
| NMC-36 | 1822 | 17.75 | -0.85 | -1.87 | -2.74 | -3.85 | 0.12 | 0.06 | 0.16 | 24.4 | 6.0* | 1 |
| NMC-38 | 1814 | 18.75 | -0.72 | -1.76 | -2.57 | -3.64 | 0.20 | 0.07 | 0.17 | 24.3 | 6.6* | 1.2 |
| NMC-40 | 1800 | 20.5 | -0.92 | -2.10 | -3.09 | -4.29 | 0.16 | 0.06 | 0.14 | 20.6 | 5.7* | 1 |

*Kang, et al. Atmospheric mercury depositional chronology reconstructed from lake sediment and ice cores in Himalaya-Tibet. *submitted*

**Table S2** Operating parameters of the Neptune Plus MC-ICP-MS.

| **Plasma parameters** | |
| --- | --- |
| Sample gas | 0.91 L min^-1^ |
| Additional gas | 0.24 L min^-1^ |
| Cool gas | 16.0 L min^-1^ |
| Auxiliary gas | 0.80 L min^-1^ |
| RF power | 1400 W |
| **Apex-Q** |  |
| Heater temperature | 100°C |
| Chiller temperature | 2°C |
| **Gas phase separator** |  |
| Solution uptake rate | 0.75 mL min^-1^ |

**Table S3** Isotopic composition of Hg for UM-Almadén

|  | Hg concentration | δ^199^Hg | σ | δ^200Hg^ | σ | δ^201Hg^ | σ | δ^202Hg^ | σ | Δ^199^Hg | σ | Δ^200Hg^ | σ | Δ^201Hg^ | σ |
| --- | --- | --- | --- | --- | --- | --- | --- | --- | --- | --- | --- | --- | --- | --- | --- |
|  | ng mL^-1^ | ‰ | ‰ | ‰ | ‰ | ‰ | ‰ | ‰ | ‰ | ‰ | ‰ | ‰ | ‰ | ‰ | ‰ |
| UM-Almadén | 0.4 | -0.12 |  | -0.21 |  | -0.37 |  | -0.52 |  | 0.01 |  | 0.05 |  | 0.02 |  |
| UM-Almadén | 0.4 | -0.18 |  | -0.29 |  | -0.45 |  | -0.55 |  | -0.04 |  | -0.01 |  | -0.04 |  |
| UM-Almadén | 0.4 | -0.11 |  | -0.26 |  | -0.4 |  | -0.46 |  | 0.01 |  | -0.03 |  | -0.05 |  |
|  |  | -0.14 | 0.04 | -0.25 | 0.04 | -0.41 | 0.04 | -0.51 | 0.05 | -0.01 | 0.03 | 000 | 0.04 | -0.02 | 0.04 |
| UM-Almadén | 0.5 | -0.14 |  | -0.22 |  | -0.37 |  | -0.45 |  | -0.03 |  | 0.01 |  | -0.03 |  |
| UM-Almadén | 0.5 | -0.18 |  | -0.23 |  | -0.41 |  | -0.53 |  | -0.05 |  | 0.04 |  | -0.01 |  |
| UM-Almadén | 0.5 | -0.12 |  | -0.23 |  | -0.42 |  | -0.5 |  | 0.01 |  | 0.02 |  | -0.04 |  |
|  |  | -0.15 | 0.03 | -0.22 | 0.01 | -0.4 | 0.03 | -0.49 | 0.04 | -0.02 | 0.03 | 0.02 | 0.02 | -0.03 | 0.02 |
| UM-Almadén | 1.0 | -0.16 |  | -0.21 |  | -0.37 |  | -0.45 |  | -0.05 |  | 0.02 |  | -0.03 |  |
| UM-Almadén | 1.0 | -0.16 |  | -0.28 |  | -0.45 |  | -0.53 |  | -0.03 |  | -0.01 |  | -0.05 |  |
| UM-Almadén | 1.0 | -0.19 |  | -0.21 |  | -0.43 |  | -0.52 |  | -0.06 |  | 0.05 |  | -0.04 |  |
|  |  | -0.17 | 0.02 | -0.23 | 0.04 | -0.42 | 0.04 | -0.5 | 0.04 | -0.05 | 0.02 | 0.02 | 0.03 | -0.04 | 0.01 |
